# Optical skill-assist device for ultrasound-guided vascular access: A preliminary simulation study

**DOI:** 10.1097/MD.0000000000016126

**Published:** 2019-06-28

**Authors:** Takayuki Asao, Mami Kikuchi, Joho Tokumine, Hisao Matsushima, Hideaki Andoh, Kazumi Tanaka, Masafumi Kanamoto, Yuki Ideno

**Affiliations:** a Gunma University Center for Mathematics and Data Science; b Center of Regional Medical Research and Education, Gunma University Hospital, Maebashi, Gunma; c Department of Anesthesiology, Kyorin University School of Medicine, Mitaka, Tokyo; d Emergency and Critical Care Center, Dokkyo Medical University Saitama Medical Center, Saitama; e Akita University Hospital Medical Simulation Center, Akita-City, Akita; f Medical Quality and Safety Management Center, Gunma University Hospital; g Intensive Care Unit, Gunma University Hospital, Maebashi, Gunma, Japan.

**Keywords:** in-plane approach, needle guide, simulation training, skill-assist, ultrasound-guided central venous catheterization

## Abstract

Ultrasound-guided central venous catheterization may cause lethal mechanical complications intraoperatively. We developed a novel device to prevent such complications. It works as a needle guide to supplement the operator's skill. We evaluated the utility of this device in terms of the success rate and visualization of the needle tip while penetrating the target vessel using a simulator.

This study was approved by the local ethics committee. The new device – an optical skill-assist device – has a slit and a mirror in the center. The operator can see the needle's reflection in the mirror through the slit and can thus ensure that the needle is directed in line with the ultrasound beam. Participants were recruited by placing an advertisement for a hands-on seminar on ultrasound-guided vascular access. They received hands-on training on the in-plane approach for 2 hours. Pre-test and post-test without the device and an additional test using the device were performed to evaluate the proficiency of ultrasound-guided vascular access. An endoscope inserted into the simulated vessel was used to detect the precise location of the needle tip in the vessel.

The primary outcomes were the success rate of the procedure. The secondary outcome was visualization of the needle tip while penetrating the simulated vessel. The chi-squared test was used for comparing the success rate and needle tip visualization between the different tests. *P* < .05 was considered to indicate significant differences.

Forty-two participants were enrolled in this study. The success rate did not increase after the simulation training (*P* = .1). Using the optical skill-assist device, the rate improved to 100%. There was a significant difference in success rate between the pre-test and additional test using the optical skill-assist device (*P* = .003). Needle tip visualization significantly improved with the use of the optical skill-assist device compared to the pre-test (*P* < .001) and post-test (*P* = .001).

Simulation training improved participants’ skill for ultrasound-guided vascular access, but the improvement depended on each participant. However, further, improvement was achieved with the use of the optical skill-assist device.

The optical skill-assist device is useful for supplementing the operator's skill for ultrasound-guided central venous catheterization.

## 1 Introduction

Ultrasound-guided central venous catheterization is defined as a technique for central venous catheterization using ultrasound guidance. In this technique, ultrasound is used for visualizing the target vein and needle in a real-time manner. Some needle guides for ultrasound-guided central venous catheterization are commercially available. The role of the needle guide is to keep the trajectory of the needle in the path of the ultrasound beam. Using the needle guide, the dependence on the operator's skill in using the in-plane approach is greatly reduced.^[1]^ Hence, a high success rate can be achieved even by novice operators.^[2]^ In other words, the learning process for novice operators can be accelerated using the needle guide.^[2,3]^ In addition, the use of the needle guide also contributes to patient safety.

However, use of the needle guide also has a disadvantage in that it prevents novice operators from acquiring skill in handling the needle in a free-hand manner, which they might have to do frequently because the use of the needle guide is still not very common in clinical practice. Since ultrasound-guided central venous access is currently considered to be the gold standard for central venous catheterization, novice operators should be encouraged to gain proficiency in performing it in a free-hand manner without depending on the support of the needle guide.

Most needle guides can be classified as mechanical needle guides. They have a slit or a groove to make the needle proceed straight and/or shoot a target.^[1–3]^ There is a specific type of needle guide that is not a mechanical needle guide. This is a “laser guide,” in which a laser beam is projected in the same plane as the ultrasound beam.^[4]^ Therefore, the laser beam indicates the plane of the ultrasound beam. The operator can recognize the laser beam as a virtual plane instead of the unseen ultrasound beam. To maintain the needle in the correct path, the entire procedure can be performed by using the laser guide. The developers of the laser guide claimed that laser assistance could help operators improve their “hand-eye coordination.” Once the operator acquires sufficient hand-eye coordination with continuous use of the laser guide, the laser beam can be ignored and the operator can perform the procedure without laser assistance. Though we agree that the laser guide may be helpful in improving operators’ hand-eye coordination, it is unclear how to ignore the laser beam during the procedure.

We have developed a new type of needle guide for use during the in-plane approach of ultrasound-guided vascular access; it can be used as a needle guide as well as a skill-assist device, depending on the situation. In this simulation study, we investigated the utility of this device as a skill-assist device for ultrasound-guided vascular access.

## 2 Methods

This study was approved by the local ethics committee of Gunma University (IRB1554). The study was planned as a single-group comparison study, and the participants were recruited by placing an advertisement for a hands-on seminar on ultrasound-guided vascular access. They received hands-on training on the in-plane approach for 2 hours. Pre-test and post-test proficiency of ultrasound-guided vascular access without the device and an additional test using the device were evaluated. An endoscope inserted into the simulated vessel was used to detect the precise location of the needle tip in the vessel.

The primary outcomes were the success rate of the procedure. The secondary outcome was visualization of the needle tip while penetrating the simulated vessel.

### 2.1 Optical skill-assist device

**Figure 1:**
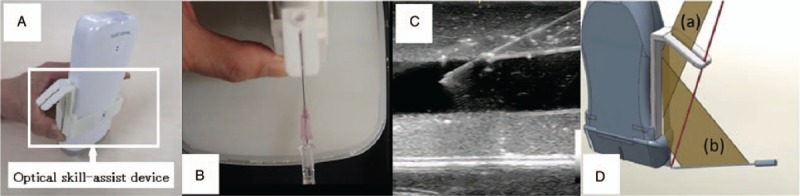
Optical skill-assist device. Panel A: Optical skill-assist device attached on ultrasound probe. Panel B: When the operator can see the needle's reflection in the mirror through the slit, the needle can be maintained in the path of the ultrasound beam (Panel C). Panel C: Ultrasound image of the needle proceeding along the path of the ultrasound beam. Panel D: Plane (a) represents the operator's sight, and plane (b) represents the needle's reflection in the mirror through the slit. The line of sight and needle's reflection are coincident in the same plane, which is the plane of the ultrasound beam.

We have invented a new skill-assist device for ultrasound-guided vascular access. We made a prototype of the device using a 3D printer, and named it the optical skill-assist device based on its function. Now, this device is sold commercially (True Puncture, Japanese Organization for Medical Device Development, Inc, Tokyo, Japan). The device has a slit and a mirror in the center (Fig. 1). The operator can watch the reflection of the needle in the mirror through the slit only when the needle, the mirror, and the operator's dominant eye are in the same plane. This means that the needle is placed in the path of the ultrasound beam and is directed in the same direction. We refer to this device as an optical skill-assist device. This device does not fix the needle in place; the needle has to be adjusted by the operator during the procedure. Therefore, this device is not a mechanical needle guide; rather, it works in a manner similar to the laser guide. However, this device can be used depending on the operator's need. Some operators may use the optical skill-assist device during all procedures. In this case, the device works as a needle guide. Others may use it only when the needle tip is not clearly visualized, in which case, the device works as a skill-assist device.

### 2.2 Needle and ultrasound machine

In this study, we used a cannula over the needles (22G, 32m, Surflo I.V. catheter; Terumo Co, Tokyo, Japan) and ultrasound machines (Isono, Alfabio Co, Japan) equipped with 10-MHz linear probes.

### 2.3 Experiment

We acquired endoscopic and ultrasound images using a computer while the participants performed the vessel puncture procedure. A simulator that simulated the internal jugular vein (inner diameter, 6 mm) was used. The participants received a hands-on training on the in-plane approach for ultrasound-guided vascular access for 2 hours. Pre-test and post-test were performed for evaluating the operators’ proficiency in ultrasound-guided vascular access. After the post-test, the participants were instructed on the use of the optical skill-assist device. The participants were cautioned on using it only when needing the assistance of the device.

### 2.4 Needle visualization

**Figure 2:**
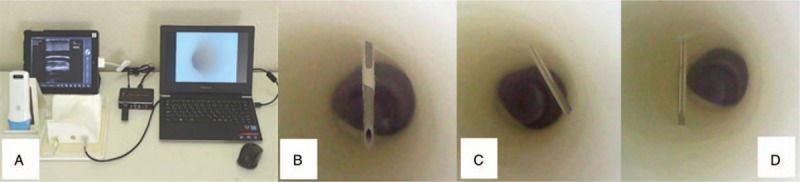
Recording system for endoscopic view in the simulated vessel. Panel A: Endoscopic view and ultrasound view are captured and saved in the computer simultaneously. Panel B: Image of a successful puncture. The needle tip is in the center of the simulated vessel. Panel C: Image of a failed puncture; lateral wall penetration. Panel D: Image of a failed puncture; posterior wall penetration.

**Figure 3:**
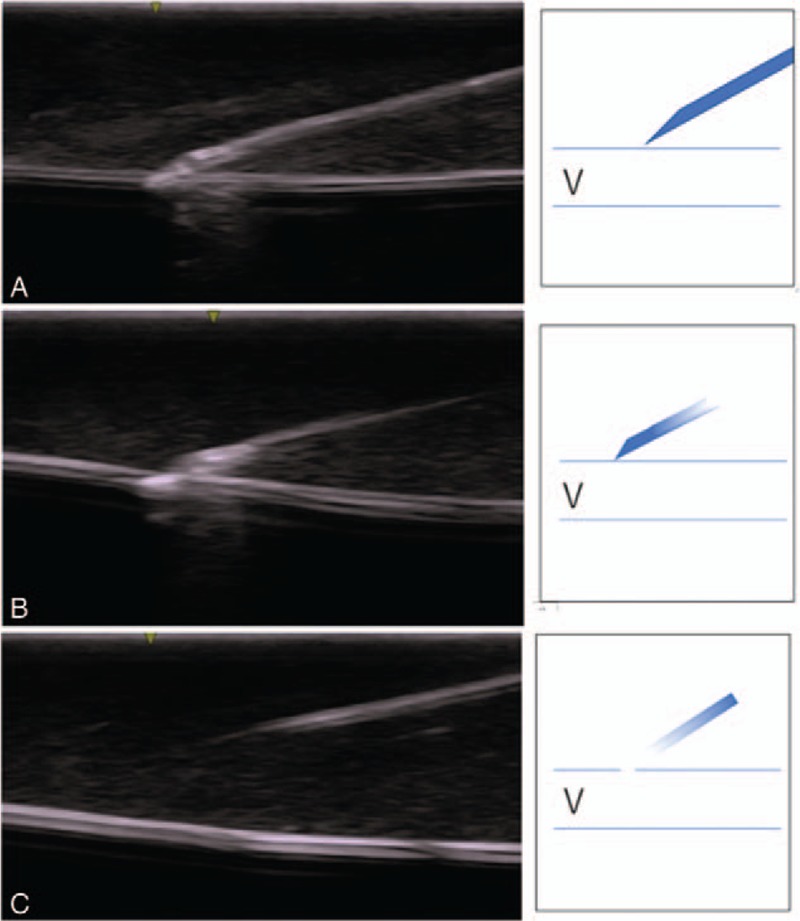
Needle tip visualization. Ultrasound experts observed the recorded ultrasound views and evaluated whether the needle tip could be classified as “identified” (clearly visible, or visible) or not (“unidentified”). Typical images judged as clearly visible, visible, and unidentified are shown above. Panel A: Clearly visible: Needle tip and shaft can be seen in full length. Panel B: Visible: Needle tip and shaft can be seen partially. Panel C: Unidentified: Needle tip cannot be observed. The shaft can be seen partially.

Ultrasound images were obtained during the puncture using an image recording system (Alfabio Co; Fig. 2). Two ultrasound experts, who did not participate in the seminars, observed the recorded view and classified it as “identified” (clearly visible or visible) or “unidentified” (Fig. 3).

### 2.5 Statistical analysis

Statistical analyses were performed using JMP Statistical Discovery (ver. 12.2.2., SAS Institute Japan Ltd, Tokyo, Japan). A *P* value <.05 was considered statistically significant.

## 3 Result

We held 5 seminars for ultrasound-guided vascular access. Informed written consent was obtained from all participants. In total, there were 42 participants, including 15 senior doctors and 27 residents. Their clinical experience ranged from <1 year to 28 years (median: 1 year). The number of previously performed ultrasound-guided central venous catheterization procedures was ≥30 for senior doctors and 1 or 2 for residents.

There was no significant difference in success rate (Table 1) as well as needle tip visualization (Table 2) between the pre-training and post-training tests. The success rate was 80.9% before the training, 92.9% after the training, and 100% with the use of the optical skill-assist device. There was no significant difference between the success rate after training and that with the use of the optical skill-assist device (Table 1). In contrast, needle tip visualization was found to be better with the use of the optical skill-assist device than that in the pre-test (*P *< .001) as well as post-test (*P* = .001; Table 2).

**Table 1:**
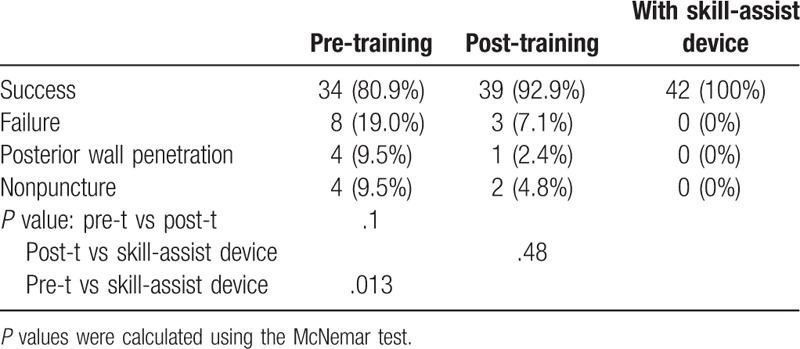
Success and failure rates.

**Table 2:**
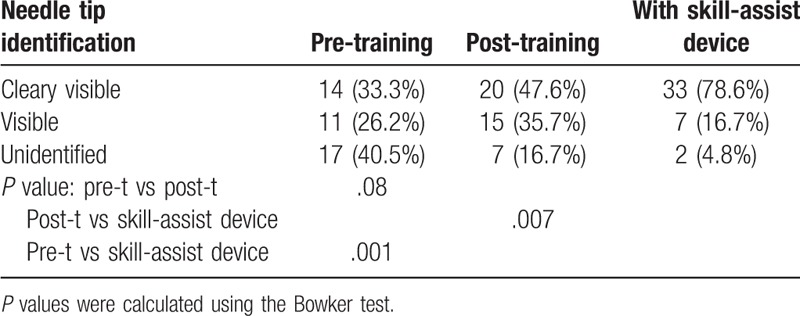
Needle tip visualization.

## 4 Discussion

The simulation training improved proficiency in some participants, but not in all. However, the optical skill-assist device could supplement the participants’ skill and thus facilitate a higher success rate. We believe that this device can help operators achieve expertise in ultrasound-guided central venous access and may also be useful in the clinical setting.

However, it is not yet known what level of proficiency is needed for safe ultrasound-guided central venous catheterization in the clinical setting. We used a difficult venous access model in this study, in which the simulated vessel was smaller than the usual size of the internal jugular vein. However, there may be other difficulties in the clinical setting, for example, twittering, collapsed veins or veins overlapped with an artery. This is a limitation of our study. Hence, a future study involving hands-on simulation training using several types of difficult venous access models is warranted. Furthermore, we should develop an educational method for safe ultrasound-guided central venous catheterization to obtain enough proficiency during difficult venous access procedures in the clinical setting.

In this study, the participants used the optical skill-assist device depending on their skill level. Some participants used the device throughout all procedures, while others used it often, sometimes, or not at all. It was observed that even participants with sufficient proficiency used the optical skill-assist device to perform the procedure perfectly. Thus, this device can be used depending on the operator's own will, and was developed with an aim to supplement the operator's skill. Further study will be needed to confirm the efficiency of this device in the clinical setting.

## 5 Conclusion

The optical skill-assist device is likely to be useful for supporting the operator's skill during ultrasound-guided central venous catheterization.

## Author contributions

**Conceptualization:** Joho Tokumine.

**Data curation:** Kazumi Tanaka.

**Project administration:** Takayuki Asao, Mami Kikuchi, Hideaki Andoh, Kazumi Tanaka, Masafumi Kanamoto.

**Supervision:** Hisao Matsushima.

**Validation:** Yuki Ideno.

**Writing – original draft:** Mami Kikuchi.

**Writing – review and editing:** Joho Tokumine.
